# Comparison of Two Massively Parallel Sequencing Platforms using 83 Single Nucleotide Polymorphisms for Human Identification

**DOI:** 10.1038/s41598-017-00510-3

**Published:** 2017-03-24

**Authors:** Dame Loveliness T. Apaga, Sheila E. Dennis, Jazelyn M. Salvador, Gayvelline C. Calacal, Maria Corazon A. De Ungria

**Affiliations:** 1 Program on Forensics and Ethnicity, Philippine Genome Center, University of the Philippines, Diliman, Quezon City, 1101 Philippines; 2 DNA Analysis Laboratory, Natural Sciences Research Institute, University of the Philippines, Diliman, Quezon City, 1101 Philippines; 3 Philippine Council for Industry, Energy and Emerging Technology Research and Development Department of Science and Technology, Bicutan, Taguig City, 1631 Philippines

## Abstract

The potential of Massively Parallel Sequencing (MPS) technology to vastly expand the capabilities of human identification led to the emergence of different MPS platforms that use forensically relevant genetic markers. Two of the MPS platforms that are currently available are the MiSeq^®^ FGx™ Forensic Genomics System (Illumina) and the HID-Ion Personal Genome Machine (PGM)™ (Thermo Fisher Scientific). These are coupled with the ForenSeq™ DNA Signature Prep kit (Illumina) and the HID-Ion AmpliSeq™ Identity Panel (Thermo Fisher Scientific), respectively. In this study, we compared the genotyping performance of the two MPS systems based on 83 SNP markers that are present in both MPS marker panels. Results show that MiSeq^®^ FGx™ has greater sample-to-sample variation than the HID-Ion PGM™ in terms of read counts for all the 83 SNP markers. Allele coverage ratio (ACR) values show generally balanced heterozygous reads for both platforms. Two and four SNP markers from the MiSeq^®^ FGx™ and HID-Ion PGM™, respectively, have average ACR values lower than the recommended value of 0.67. Comparison of genotype calls showed 99.7% concordance between the two platforms.

## Introduction

Massively parallel sequencing (MPS) has undoubtedly taken DNA-based analysis to a higher level of exploration and discovery. In forensic sciences, MPS surpassed conventional DNA profiling technologies, e.g. capillary electrophoresis-based sequencing, because 1) MPS is capable of obtaining detailed sequence information on conventional genetic markers; 2) MPS has increased multiplexing capabilities; 3) MPS reports massive amounts of data simultaneously from one or many individuals; and 4) MPS provides a higher throughput DNA sequencing procedure^1, 2^. Several commercial kits that use the MPS platforms for forensic STR^3–5^ and mtDNA^6, 7^ analyses are now available. MPS has also boosted the power of SNP markers for human identification by increasing the number of SNP loci that are analyzed in one reaction. Before MPS, SNP typing was performed using single base extension (SBE) reaction. This produced SNP typing procedures that are capable of multiplexing around 50 SNP loci. On its own, human identification via SNP markers wasn’t able to gain as much popularity as STRs and mtDNA markers in the forensic community^8–10^. Since the inception of MPS in forensic science, Illumina^®^^11^ and Life Technologies™^12–14^ developed marker panels with more than 100 SNP loci. The MPS platforms dedicated for forensic genetics are the MiSeq^®^ FGx™ Forensic Genomics System (Illumina) and the HID-Ion Personal Genome Machine (PGM)™ (Thermo Fisher Scientific). These platforms are coupled with the commercially available panels for human identification, the ForenSeq™ DNA Signature Prep kit (Illumina) and the HID-Ion AmpliSeq™ Identity Panel (Thermo Fisher Scientific). These MPS systems sequence in one reaction 124 (HID-Ion PGM™) and 173 (MiSeq^®^ FGx™, with additional 59 STR markers) SNP markers, respectively. 83 of these SNP markers are common for both platforms. These 83 SNP markers, which are spread across the 22 autosomes, have high heterozygosity and low Fixation Index (Fst) giving them a high combined discrimination power^15, 16^. In addition, these 83 SNPs have relatively small amplicon sizes ranging from 40 to 135 bp increasing the likelihood of successful DNA profiling of degraded DNA^17, 18^.

Since HID-Ion PGM™ and MiSeq^®^ FGx™ systems utilize different approaches to sequencing, it is necessary to assess the reliability and consistency of their genotyping results. Concordance of the shared 83 SNP markers will allow merging of data that were generated by the two systems and enable expansion of existing databases. Using the overlapping 83 SNPs, we performed concordance analysis and parallel evaluation in terms of coverage per SNP locus and heterozygote balance of genotype calls on 143 blood samples that were blotted on FTA™ paper (Whatman). FTA™ paper is used in forensics genetics research and biobanking because it allows for easier and longer storage of DNA from samples such as blood^19^. Recently, Kampmann and co-workers demonstrated the utility of FTA samples for MPS^20^ thus opening the doors for laboratories with archived samples to adopt the MPS technology.

## Results and Discussion

### Concordance Evaluation

**Figure 1:**
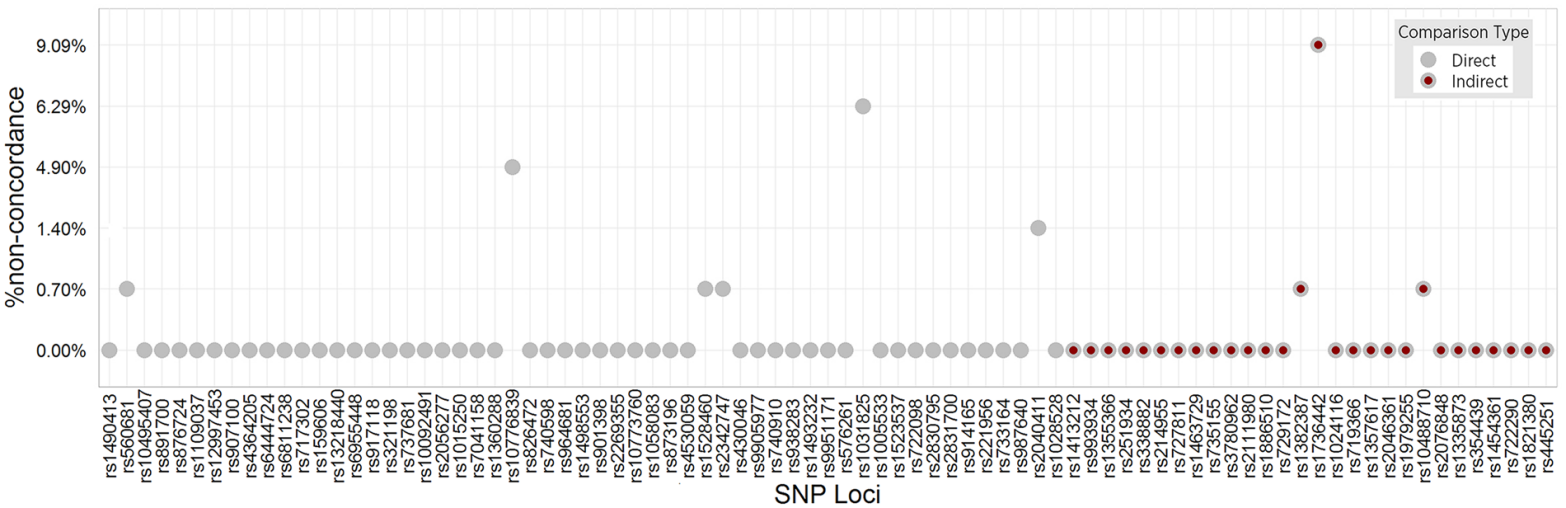
Percentage of non-concordance between the HID-Ion PGM™ and MiSeq^®^ FGx™ MPS platforms. Direct Comparison: Concordance evaluation was performed using raw genotype calls reported by the MPS platforms. Indirect Comparison: Concordance evaluation was performed after reverse complementation of all observed genotypes at the 28 SNP loci from the MiSeq^®^ FGx™ platform. Reverse complementation was necessary because the two MPS systems use different nomenclature in reporting SNP calls.

**Figure 2:**
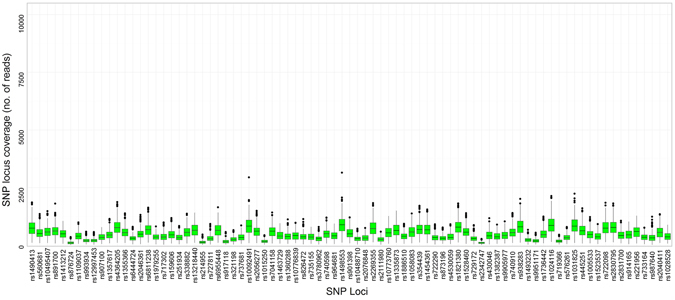
Distribution of read counts and median coverage of the SNP markers typed with HID-Ion PGM™.

**Figure 3:**
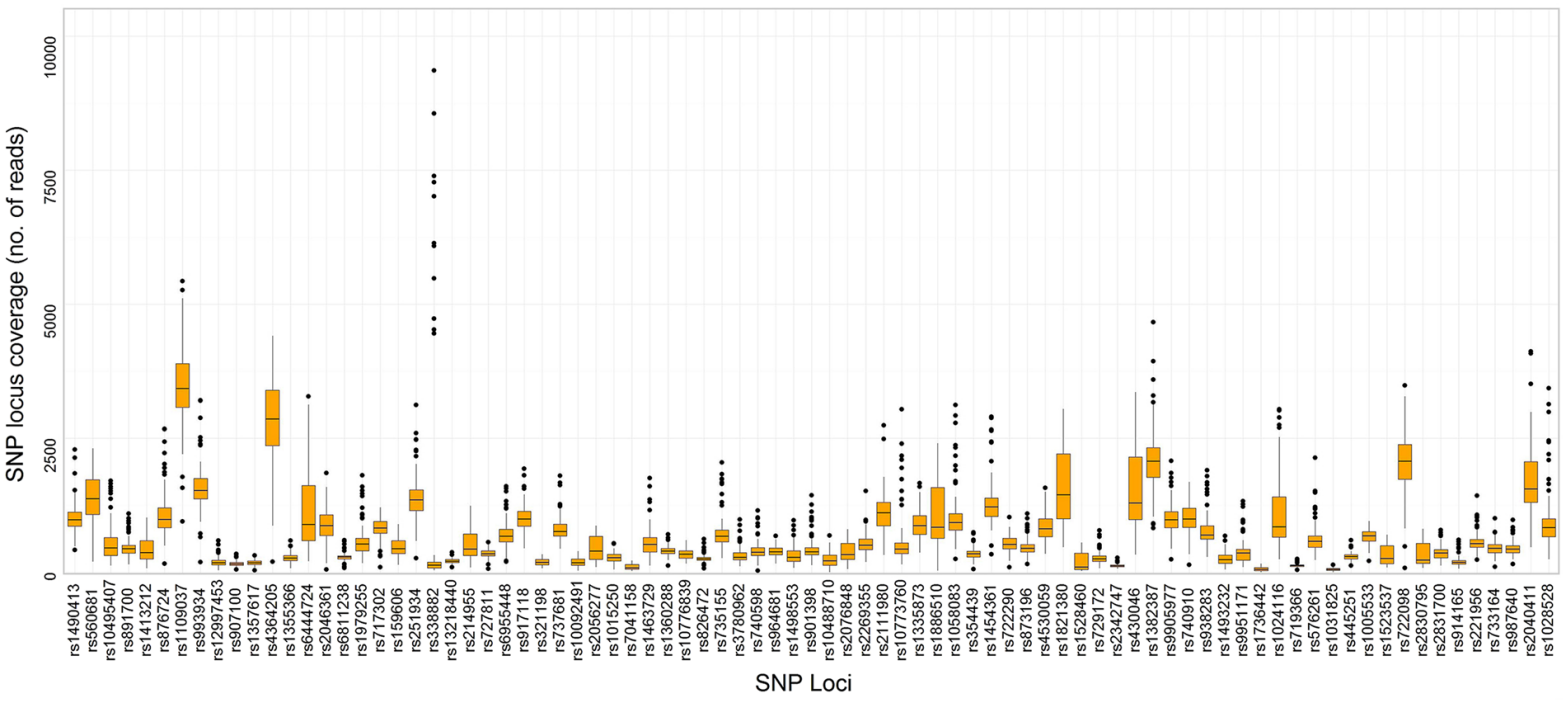
Distribution of read counts and median coverage of the SNP markers typed with MiSeq^®^ FGx™ Forensic Genomics System.

The two MPS systems initially showed more than 43% non-concordance at 28 out of the 83 SNPs analyzed (Fig. 1, gray circle with red dot) because the two MPS platforms use different nomenclature in reporting SNP genotypes. Concordance was achieved after comparing the reverse complement of the genotypes from MiSeq^®^ FGx™ marker panel with the corresponding genotypes in the HID-Ion PGM™ marker panel. Overall concordance analysis of the 143 samples showed an average of 99.70% concordance and a non-concordance range of 0 to 9% across all the 83 SNPs (Fig. 1). Non-concordance was contributed mainly by zero or low coverage reads (Figs 2 and 3) and extreme allele imbalance (Table 1). Multiple samples exhibited non-concordance at SNPs rs1736442 (9%), rs1031825 (6%), and rs10776839 (5%) (Table 1). SNPs rs1736442 and rs1031825 showed low average coverage reads of 58 and 54 (Fig. 3), respectively, when typed with MiSeq^®^ FGx™ platform. For such cases, the risk of allele dropout is higher because of the low number of allele reads^1^. SNPs rs10776839 and rs2040411 gave very low or imbalanced average allele coverage ratio (ACR) values of 0.186 and 0.097, respectively, for the samples listed in Table 1 when typed in HID-Ion PGM™ system. Notably, SNP rs10776839 is among the poorly performing SNPs identified for Ion Torrent™ HID SNP assay due to inconsistent allele balance among samples typed^1^.

**Table 1:** Comparison of genotype calls from the HID-Ion PGM™ and MiSeq^®^ FGx™.

dbSNP ID	Sample No.	HID Ion			MiSeq FGx		
		Genotype (reads)		ACR	Genotype (reads)		ACR
rs1736442	1	C (255)	T (239)	0.937	C (12)	T (0)	n/a
rs1736442	4	C (88)	T (86)	0.977	C (0)	T (12)	n/a
rs1736442	6	C (625)	T (619)	0.990	C (14)	T (0)	n/a
rs1736442	104	C (215)	T (214)	0.995	C (12)	T (0)	n/a
rs1736442	11	C (265)	T (263)	0.992	C (0)	T (17)	n/a
rs1736442	17	C (330)	T (364)	0.907	C (0)	T (17)	n/a
rs1736442	18	C (197)	T (184)	0.934	C (0)	T (17)	n/a
rs1736442	20	C (272)	T (261)	0.960	C (0)	T (17)	n/a
rs1736442	32	C (237))	T (249)	0.952	C (22)	T (0)	n/a
rs1736442	46	C (366)	T (378)	0.968	C (15)	T (0)	n/a
rs1736442	72	C (140)	T (124)	0.886	C (0)	T (11)	n/a
rs1736442	87	C (342)	T (367)	0.932	C (24)	T (0)	n/a
rs1736442	21	C (162)	T (172)	0.942	C (0)	T (0)	n/a
rs1031825	2	A (1171)	C (1066)	0.91	A (0)	C (11)	n/a
rs1031825	3	A (909	C (841)	0.925	A (0)	C (15)	n/a
rs1031825	5	A (288)	C (278)	0.965	A (13)	C (0)	n/a
rs1031825	57	A (221)	C (203)	0.919	A (23)	C (0)	n/a
rs1031825	58	A (326)	C (298)	0.914	A (12)	C (0)	n/a
rs1031825	82	A (388)	C (354)	0.912	A (0)	C (22)	n/a
rs1031825	93	A (468)	C (425)	0.908	A (0)	C (14)	n/a
rs1031825	6	A (908)	C (839)	0.924	A (0)	C (0)	n/a
rs1031825	53	A (525)	C (443)	0.844	A (0)	C (0)	n/a
rs10776839	1	G (62)	T (347)	0.179	G(0)	T (253)	n/a
rs10776839	21	G (176)	T (765)	0.230	G(0)	T (172)	n/a
rs10776839	33	G (109)	T (608)	0.179	G(0)	T (332)	n/a
rs10776839	44	G (131)	T (604)	0.217	G(0)	T (402)	n/a
rs10776839	56	G (330)	T (1601)	0.206	G(0)	T (326)	n/a
rs10776839	65	G(124)	T (788)	0.157	G(0)	T (286)	n/a
rs10776839	79	G (135)	T (1037)	0.130	G(0)	T (322)	n/a
rs2040411	32	A (112)	G (1103)	0.102	A (943)	G (1000)	0.943
rs2040411	120	A (30)	G (327)	0.092	A (416)	G (397)	0.954
rs560681	63	A (262)	G (248)	0.947	A (12)	G (826)	0.015
rs10488710	6	C (300)	G (304)	0.987	C (0)	G (0)	n/a
rs1528460	19	C (621)	T (592)	0.953	C (0)	T (22)	n/a
rs2342747	54	A (112)	G (119)	0.941	A (83)	G (26)	0.313
rs1382387	130	A (169)	C (192)	0.880	A (55)	C (1095)	0.050

### Coverage Analysis

Sequencing coverage directly affects the sensitivity and SNP genotyping accuracy of MPS systems applied to forensics typing. For SNP detection, the actual coverage per SNP locus (referred to as ‘SNP coverage’ in this paper) was the parameter used for evaluation^21^. For HID-Ion PGM™ and MiSeq^®^ FGx™, the variation in read counts could be brought by varying factors during library preparation. SNP coverage for HID-Ion is affected by the number of wells in the sequencing chip which are occupied by Ion Sphere™ Particles (ISP) with monoclonally amplified SNP target that were successfully read^21^. On the other hand, SNP coverage for the MiSeq^®^ FGx™ is affected by the PCR amplification efficiency, purification, and bead-based library normalization^11^. Increased number of markers multiplexed in one sequencing reaction could also increase variation in coverage of the SNP markers^11^. Comparison of the markers’ SNP coverage between the two MPS systems (Figs 2 and 3) showed that MiSeq^®^ FGx™ achieved higher SNP coverage reads in majority of the markers; however, it also showed higher variation in SNP coverage distribution across samples than the HID-Ion PGM™, with more read outliers observed in the univariate statistical evaluation. In MiSeq^®^ FGx™, SNP rs338882 (average ACR = 0.51) gave 11 extreme outliers with SNP coverage values that are at most 8,740 reads away from the average SNP coverage (618 reads) of the 143 samples (Fig. 3). This SNP, however, showed 100% concordance between platforms.

### Allele Coverage Ratio

**Figure 4:**
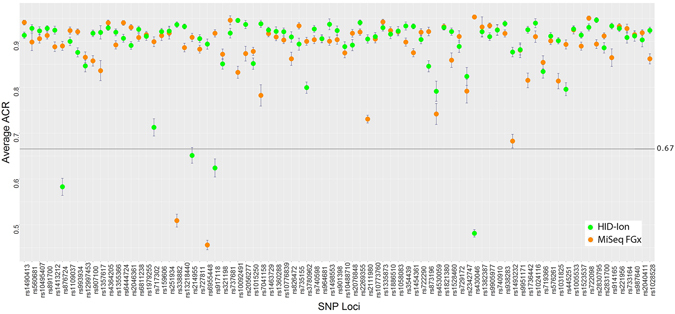
Allele Coverage Ratio of SNPs from the HID-Ion PGM™ and the MiSeq^®^ FGx™ MPS platforms. Gray line marks the 0.67 ACR threshold for balanced heterozygous alleles.

The over all average allele coverage ratio (ACR) of heterozygous SNPs is 0.89 and 0.88 for HID-Ion PGM™ and MiSeq^®^ FGx™ platforms (Fig. 4), respectively. This means that coverage of heterozygous SNPs on the two platforms is generally balanced approximating the ideal ACR value of 1.0 (50:50 allele ratio). The SNPs rs214955, rs430046, rs876724, and rs917118, in the HID-Ion PGM™, and SNPs rs338882 and rs6955448, in the MiSeq^®^ FGx™ (Fig. 4), gave average ACR values of less than 0.67, which was the recommended minimum threshold value of Eduardoff *et al*. for balanced heterozygote SNPs^21^. This, however, did not affect concordance between platforms of the SNPs mentioned.

## Conclusion

The study puts forward the need to include the information on sequence nomenclature when reporting MPS data. Genotyping data generated using HID-Ion PGM™ and MiSeq^®^ FGx™ Forensic Genomics System were highly concordant and SNP data may be pooled to provide a more comprehensive database of forensically relevant SNPs. Further work is needed to address the quality of MPS data from the SNPs rs10776839, rs1031825 and rs1736442 – with greater than 4.8% non-concordance between platforms– and the SNP rs338882 in MiSeq^®^ FGx™ marker panel– with observed imbalance in heterozygous SNPs and with large sample-to-sample coverage read variation.

## Methods

The study was implemented at the DNA Analysis Laboratory, Natural Sciences Research Institute, University of the Philippines Diliman. Laboratory work involving the use of MPS machines was conducted at Illumina Headquarters in Singapore and at the Philippine Genome Center, University of the Philippines Diliman. Steps in processing the samples using the HID-Ion PGM™ and MiSeq^®^ FGx™ ForenSeq™ Genomics System were performed following the manufacturers’ protocols^22–24^.

### Samples

Archived blood DNA samples on FTA™ paper from 143 unrelated Filipino male individuals were processed using the MiSeq^®^ FGx™ Forensic Genomic System and the HID-Ion PGM™ following manufacturers’ protocols. This study was approved by the University of the Philippines Manila, Research Ethics Board (UPMREB No. 2014-499-01). All procedures were performed in accordance with the approved guidelines of UPMREB. Volunteer’s informed consent was obtained before sample collection was performed.

### SNP markers

The 83 overlapping autosomal Individual Identification SNPs are composed of 37 SNPs reported by Ken Kidd^15^ and 47 SNPS used by the SNPforID Consortium^16^, with one common SNP (dbSNP ID rs2046361). Primers targeting these SNPs are included in the HID-Ion AmpliSeq™ Identity Panel (Thermo Fisher) and ForenSeq™ DNA Signature Prep Kit Primer Mix B (Illumina). HID-Ion AmpliSeq™ Identity Panel is composed of the 34 Y-clade SNPs and 90 autosomal SNPs. ForenSeq™ DNA Signature Prep Kit Primer Mix B (Illumina) is composed of 95 Identity Informative SNPs, 22 Phenotypic Informative SNPs, and 56 Ancestry Informative SNPs^22^.

### Massively Parallel Sequencing using HID-Ion PGM™

Library amplification was performed in the Veriti^®^ Thermal Cycler (Applied Biosystems) using reagents from the HID Ion AmpliSeq™ Identity Panel kit^24^. The amplification reaction contained 5X Ion AmpliSeq™ HiFi Master Mix (4 ul) and 2X HID-Ion AmpliSeq™ Identity SNP-124 Panel (10 ul). PCR cycling was reduced to 18 cycles for FTA™ discs. Partial digestion of primers was performed by treating the amplicons with FuPa Reagent (2 ul) (Thermo Fisher Scientific). This was followed by the ligation of barcodes to the amplicons using Switch Solution (4 ul), DNA ligase (2 ul), and Ion Xpress Barcode (2 ul) (Thermo Fisher Scientific). Barcoded libraries were purified with Agencourt AMPure XP Reagents (Beckman Coulter, Brea, CA) and quantified using the Ion Library TaqMan PCR Mix (Thermo Fisher Scientific) and the 20X Quantitation Assay (Thermo Fisher Scientific). Pooling of the library was performed by mixing equal volumes of barcoded samples with a concentration of 20 pM. Clonal amplification via emulsion PCR and library enrichment was performed using the Ion OneTouch™ 2 (OT2) System with the Ion PGM™ Template OT2 200 Kit and the Ion OneTouch™ ES (Thermo Fisher Scientific). Sequencing was performed in the Ion Torrent PGM™ (Thermo Fisher Scientific) with Ion PGM™ Sequencing 200 Kit and the Ion 318 Chip Kit v2 (Thermo Fisher Scientific) following manufacturer’s protocol.

### Massively Parallel Sequencing using MiSeq^®^ FGx™ Forensic Genomics System

Library amplification, amplicon indexing and barcoding were performed using the ForenSeq™ DNA Signature Prep Kit following recommended manufacturer’s protocol for FTA samples^22^. The libraries underwent a bead- based purification and normalization using the ForenSeq™ DNA Signature Prep Kit. The normalized libraries were pooled using equal volumes of preparations with 0.2 ng/ul concentration. Pooled libraries were diluted in hybridization buffer and denatured. Sequencing was performed on the MiSeq^®^ FGx™ desktop sequencer with the MiSeq^®^ ForenSeq™ Sequencing Kit (Illumina) following manufacturer’s protocol.

### Data Analysis

For the HID-Ion PGM™, raw sequencing data were processed in the Ion Torrent Suite™ Software with the HID SNP Genotyper Plugin adapted for data analysis. ForenSeq™ Universal Analysis Software (UAS) (Illumina) was used for the MiSeq^®^ FGx™ Forensic Genomics System. Genotype calls and coverage reads were exported as Microsoft^®^ Office Excel^®^ (2007) files from the HID SNP Genotyper Plugin and ForenSeq™ UAS software. Data analysis and presentation were performed in Matlab v.2 (Mathworks, Natick, MA, USA) and R software v.3.3.1 using the ggplot2 package.
